# Diversity of extracellular HSP70 in cancer: advancing from a molecular biomarker to a novel therapeutic target

**DOI:** 10.3389/fonc.2024.1388999

**Published:** 2024-04-05

**Authors:** Binbin Hu, Guihong Liu, Kejia Zhao, Gao Zhang

**Affiliations:** ^1^ Department of Radiation Oncology, Cancer Center, West China Hospital, Sichuan University, Chengdu, Sichuan, China; ^2^ Department of Thoracic Surgery and Institute of Thoracic Oncology, West China Hospital, Sichuan University, Chengdu, Sichuan, China; ^3^ Western China Collaborative Innovation Center for Early Diagnosis and Multidisciplinary Therapy of Lung Cancer, Chengdu, Sichuan, China; ^4^ Faculty of Dentistry, The University of Hong Kong, Hong Kong, Hong Kong SAR, China

**Keywords:** extracellular HSP70, cancer, biomarker, treatment, vaccine

## Abstract

Heat shock protein 70 (HSP70) is a highly conserved protein functioning as a “molecular chaperone”, which is integral to protein folding and maturation. In addition to its high expression within cells upon stressful challenges, HSP70 can be translocated to the cell membrane or released from cells in free form or within extracellular vesicles (EVs). Such trafficking of HSP70 is also present in cancer cells, as HSP70 is overexpressed in various types of patient samples across a range of common malignancies, signifying that extracellular HSP70 (eHSP70) can serve as a tumor biomarker. eHSP70 is involved in a broad range of cancer-related events, including cell proliferation and apoptosis, extracellular matrix (ECM) remodeling, epithelial-mesenchymal transition (EMT), angiogenesis, and immune response. eHSP70 can also induce cancer cell resistance to various treatments, such as chemotherapy, radiotherapy, and anti-programmed death-1 (PD-1) immunotherapy. Though the role of eHSP70 in tumors is contradictory, characterized by both pro-tumor and anti-tumor effects, eHSP70 serves as a promising target in cancer treatment. In this review, we comprehensively summarized the current knowledge about the role of eHSP70 in cancer progression and treatment resistance and discussed the feasibility of eHSP70 as a cancer biomarker and therapeutic target.

## 1 Introduction

Heat shock protein 70 (HSP70) is a crucial member of the heat shock protein family with a molecular weight of approximately 70 kD. This protein is highly conserved across different species, indicating its essential role in maintaining cellular homeostasis. The human HSP70 family has 13 homologs, among which mitochondrial HSP70 (HSPA9/GRP75/Mortalin) and endoplasmic reticulum HSP70 (HSPA5/GRP78/BiP) are the most extensively studied members (1). Despite differences in gene locus, amino acid residues, and subcellular localization, the HSP70 family has a common structure consisting of two major functional domains: a conserved nucleotide-binding domain (NBD) at N-terminal and a more variable substrate-binding domain (SBD) at C-terminal. The NBD is crucial for binding to and hydrolyzing ATP, while the SBD can bind to substrate proteins. Coupled with ATP hydrolysis, HSP70 interacts with substrate proteins, facilitating their correct folding, preventing aggregation, and refolding damaged proteins (2). Thus, HSP70 functions as a “molecular chaperone”.

Upon stressful challenges, HSP70 is highly induced and capable of directly inhibiting cellular apoptosis (3). However, in pathologic conditions like cancer, upregulated HSP70 induces disease progression and treatment resistance (4). Extensive studies have indicated that HSP70 is not only highly expressed in tumor cells but also can be released extracellularly. Notably, such a type of transport or extracellular expression is minimal in normal cells. Hence, extracellular HSP70 (eHSP70) endows with “cancer” characteristics. This character can be extended to two facets: first, it can be leveraged as a tumor biomarker for development; second, exploration of the correlation between eHSP70 and tumor progression can be undertaken to evaluate its feasibility as a molecular target for cancer therapy. Numerous researchers have robustly demonstrated the feasibility of these two approaches; however, there is no corresponding approved strategy for clinical application. In this review, we comprehensively summarized the current knowledge about the role of eHSP70 in cancer progression and treatment resistance and discussed and explored the feasibility of eHSP70 as a cancer biomarker and therapeutic target.

## 2 Translocation of eHSP70

**Figure 1:**
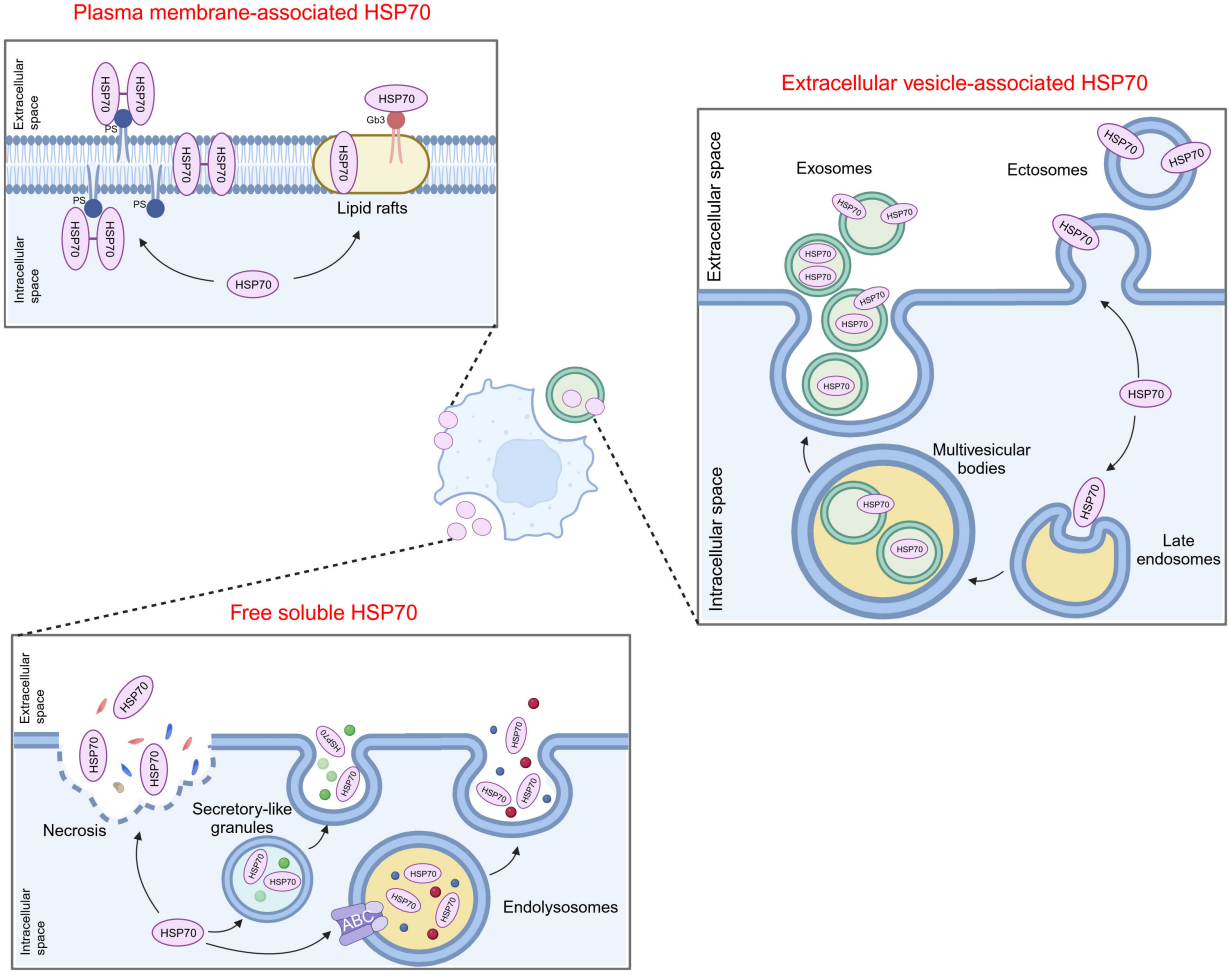
Translocation of extracellular HSP70. HSP70 can localize on the cell membrane or be transported to the extracellular milieu, either associated with extracellular vesicles or as free soluble protein. PS, phosphatidylserine; Gb3, globotriaosylceramide; ABC, ATP-binding cassette.

HSP70 can be found on the cell membrane or be transported to the extracellular milieu, either associated with extracellular vesicles (EVs) or as free soluble protein (**Figure 1**). Among the existing reports and databases, the HSP70 family members identified for expression on the plasma membrane encompass HSPA1A, HSPA1B, HSPA1L, HSPA2, HSPA5, and HSPA8, while those secreted extracellularly in the form of exosomes include HSPA1A, HSPA1B, HSPA2, GRP78, HSPA6, HSPA8, mortalin, HSPA12A, and HSPA13 (5). The association of HSP70 with the cell membrane does not seem to be simply due to the lack of discernible membrane-binding motifs or other translocation signatures within HSP70. However, extensive studies have confirmed its insertion into lipid bilayers with high affinity towards negatively charged phospholipids, particularly phosphatidylserine (PS) (6–9). HSP70 spontaneously relocates from the cytosol into the plasma membrane after oligomerization and binding to PS (10). Lipid rafts are microdomains enriched in cholesterol, glycosphingolipids, and protein receptors. It has been discovered that HSP70 preferentially localizes in lipid rafts, with one potential mechanism being facilitated via non-covalent interactions with globotriaosylceramide (Gb3) (11, 12). Besides, palmitoyl-oleoyl phosphatidylglycerol (POPG) and sulfogalactosyl ceramide, which are also negatively charged, have been reported to mediate the insertion of HSP70 into membrane structures (8, 13).

The secretion of HSP70 into the extracellular milieu also appears complex. Free soluble HSP70 was initially believed to be exclusively the result of passive release after cell death until several reports indicated that this release mainly depends on active mechanisms (14). One possible way is that HSP70 is exported via secretory-like granules (15). Additionally, Mambula and Calderwood found that HSP70 can infiltrate into endolysosomes via ATP-binding cassette (ABC) family transporter proteins and subsequently being released from cells after the fusion of endolysosomes with the plasma membrane (16). HSP70 can also be released into or onto EVs by either exocytosis of exosomes or by budding of the plasma membrane (ectosomes) (17–19). Several studies demonstrated that post-translational modifications may be involved in mediating the translocation of HSP70 into exosomes (20, 21). Moreover, oligomerized HSP70 was shown to be preferentially loaded into exosomes (22, 23).

It is noteworthy that these mechanisms are not exclusively activated in a single type of cell or under a uniform condition, and multiple eHSP70 transport modalities may concurrently exist in the same cell type. Moreover, there are many types of stimuli that induce HSP70 insertion into membrane structures or release into the extracellular space. It is evident that these translocations mostly occur in cancer cells. In response to stressful stimuli, such as hypoxia, immune response, and therapeutic stress, cancer cells may circumvent pressure by inducing the manifestation of eHSP70. Hence, advanced exploration of the extracellular transport of HSP70 and its triggering parameters can help obstruct the intrinsic protection of cancer cells at its origin.

## 3 The role of eHSP70 as a tumor biomarker

Previously, clinical studies indicated that HSP70 displays specific expression in numerous tumors across diverse types of samples, such as tumor tissues, peripheral blood, and urine, verifying its predictive role in tumor diagnosis, treatment, and prognosis (**Table 1**). Compared with healthy individuals or those suffering from benign conditions, elevated expression of circulating HSP70 is observed in the peripheral blood samples of cancer patients, and its expression is significantly amplified in the cell membranes within tumor tissues. This phenomenon spans a range of common malignancies; thus, eHSP70 can serve as a biomarker for cancer diagnosis (25, 26, 28, 29, 37, 40, 41, 43, 45, 49, 52–54, 58–63, 66, 68). Additionally, eHSP70 expression has been observed to positively correlate with the malignant behavior of tumors. Patients with advanced non-small cell lung cancer (NSCLC) exhibit elevated serum levels of HSP70 or GRP78, compared to the early-stage cases (34, 69). In breast cancer, individuals with increased serum levels of HSPA1A commonly display a higher histological grade and cell proliferation index (Ki67) (43). Given these findings, a decrease in eHSP70 levels often predict improved therapeutic responses. For instance, the concentrations of circulating HSP70 decrease as the tumor volume decreases after radiotherapy among patients with glioblastoma. Reduced expression of circulating HSP70 predicts prolonged overall survival (OS) in these patients (30, 64). It is worth noting that elevated eHSP70 levels do not necessarily imply a poorer outcome. Pfister et al. found that in patients with colon and gastric carcinomas, the expression of membrane-associated HSP70 correlates significantly with an improved OS (49). Moreover, a study also demonstrated that the concentration of circulating HSP70 significantly decreased in patients with lung cancer compared to healthy controls (26). These conclusions may originate from the differences in patient characteristics, or the paradoxical role of eHSP70 in tumor progression.

**Table 1:** Selection of studies assessing extracellular HSP70 as a potential cancer biomarker in patient samples.

Cancer type	HSP70 type	Patient sample	HSP70 localization or secretion mode	Use of eHSP70 as a biomarker	Refs
Lung cancer	HSP70	Plasma/serum	Exosomes	Diagnosis; metastasis; treatment response	(24)
			—	Diagnosis; TNM stage; recurrence	(25)
			—	Diagnosis (inverse correlation)	(26)
		Urine	Exosomes	Diagnosis	(27)
		Tumor tissue	Cell membrane	Diagnosis	(28)
	GRP78	Plasma	—	Diagnosis	(29)
NSCLC	HSP70	Plasma/serum	Free soluble forms and vesicles/exosomes	Diagnosis; radiotherapy response; gross tumor volume; TNM stage	(30–32)
			Exosomes	Radiotherapy response	(33)
			—	TNM stage	(34)
	GRP78	Serum	—	TNM stage; prognosis	(35)
	Autoantibody against HSP70	Plasma/serum	—	Diagnosis	(36)
SCLC	HSP70	Serum	—	Diagnosis; TNM stage; prognosis	(37)
ESCC	Autoantibody against HSP70	Serum	—	Diagnosis	(38)
Breast cancer	HSP70	Plasma/serum	Exosomes	Diagnosis; metastasis; treatment response	(24)
			Micro-vesicles	Treatment response	(39)
			—	Diagnosis; TNM stage; lymph node metastasis; radiotherapy response	(40–42)
		Urine	Exosomes	Diagnosis	(27)
	HSPA1A	Serum	—	Diagnosis; histological grade; Ki67 expression	(43)
	GRP78	Tumor tissue	Cell membrane	Prognosis; chemotherapy response; progesterone receptor and p53 expression	(44)
Colorectal cancer	HSP70	Serum	—	Diagnosis; TNM stage; prognosis	(45–48)
		Tumor tissue	Cell membrane	Diagnosis, prognosis (inverse correlation)	(28, 49)
	Mortalin	Serum	—	Prognosis	(50)
	Autoantibody against GRP78	Serum	—	Diagnosis	(51)
Gastric cancer	HSP70	Tumor tissue	Cell membrane	Diagnosis, prognosis (inverse correlation)	(49)
	GRP78	Serum	—	Diagnosis	(52)
	Autoantibody against GRP78	Serum	—	Diagnosis	(53)
HCC	HSP70	Serum	—	Diagnosis	(54)
	Autoantibody against GRP78	Serum	—	Diagnosis; TNM stage; portal vein invasion; metastasis	(55, 56)
Cholangiocarcinoma	Autoantibody against HSP70	Plasma	—	Diagnosis	(57)
Pancreatic cancer	HSP70	Serum	—	Diagnosis	(58)
		Tumor tissue	Cell membrane	Diagnosis	(28)
Prostate cancer	HSP70	Plasma	—	Diagnosis	(59)
Ovarian cancer	HSP70	Urine	Exosomes	Diagnosis	(27)
	GRP78	Serum	—	Diagnosis	(60)
Endometrial cancer	GRP78	Intraperitoneal fat/plasma	—	Diagnosis	(61)
RCC	GRP78	Serum	—	Diagnosis; TNM stage; histological grade	(62)
HNSCC	HSP70	Tumor tissue/serum	Cell membrane and free soluble forms	Diagnosis	(63)
Glioblastoma	HSP70	Plasma/serum	Free soluble forms and vesicles	Diagnosis; prognosis; radiotherapy response	(30, 64)
		Tumor tissue/serum	Cell membrane	Histological grade; prognosis	(65)
NPC	HSP70	Serum	—	Diagnosis; TNM stage	(66)
	Autoantibody against HSP70	Serum	—	Diagnosis	(67)
Melanoma	GRP78	Serum	Exosomes	Metastasis	(50)
AML/ALL	HSP70	Plasma	—	Diagnosis; prognosis	(53, 68)
		Tumor tissue	Cell membrane	Diagnosis	(28)
	Autoantibody against HSP70	Plasma	—	Diagnosis; prognosis	(53)
GBC	Autoantibody against HSP70	Serum	—	Prognosis	(69)
Neuronal cancer	HSP70	Tumor tissue	Cell membrane	Diagnosis	(28)

eHSP70, extracellular HSP70; NSCLC, non-small cell lung cancer; SCLC, small cell lung cancer; ESCC, esophageal squamous cell carcinoma; HCC, hepatocellular carcinoma; RCC, renal cell carcinoma; HNSCC, head and neck squamous cell carcinoma; NPC, nasopharyngeal carcinoma; AML, acute myeloid leukemia; ALL, acute lymphoblastic leukemia; GBC, cancer of the gingivobuccal complex.

Nonetheless, quantifying the levels of HSP70 in patients’ blood or other body fluids revealed inconclusive results, partly due to the short half-life of HSP70 in blood. Consequently, several studies have quantified circulating autoantibodies against HSP70. Elevated levels of autoantibodies against HSP70 have been corroborated in different cancers, including NSCLC (36), esophageal squamous cell carcinoma (38), colorectal cancer (51), gastric cancer (70), hepatocellular carcinoma (HCC) (55, 56), cholangiocarcinoma (57), nasopharyngeal carcinoma (67), acute myeloid leukemia (AML)/acute lymphoblastic leukemia (ALL) (53), and cancer of gingivobuccal complex (69). In HCC, the presence of autoantibodies against GRP78 was associated with clinical stage, portal vein invasion, and metastasis (55). These findings prove the potential of autoantibodies against HSP70 as alternative cancer biomarkers.

Surprisingly, HSP70 also exists in the form of EVs in peripheral blood or urine samples of patients with lung cancer (24, 27), NSCLC (30–33), breast cancer (24, 27, 39), ovarian cancer (27), glioblastoma (30, 64), and melanoma (50), protecting it from the phospholipid bilayer membrane and making it more stable. This discovery has thus opened up novel avenues for validating eHSP70 as an effective biomarker for cancers. The levels of exosomal HSP70 in plasma/serum samples of patients with lung cancer, breast cancer, or melanoma are correlated with tumor metastasis (24, 50). Another study showed that among 35 patients with post-therapeutic regression two years after the initial diagnosis of non-metastatic breast cancer, escalating quantities of micro-vesicular HSP70 were observed in two patients during treatment. Conversely, patients who did not succumb to a relapse maintained unchanged levels of this protein, suggesting the potential role of micro-vesicular HSP70 in predicting therapeutic efficacy (39). Moreover, Chanteloup and colleagues found that circulating exosomal HSP70 levels, but not free soluble HSP70, reflect HSP70 levels in tumor biopsies. Exosomal levels of HSP70 were more sensitive tumor dissemination predictors compared with circulating tumor cells (24). Similar to purifying circulating tumor cells (CTCs), efficacious EV isolation from diverse biological fluids is technically challenging. There are several methods for EV isolation, and the most commonly used method is differential ultracentrifugation (UC). Other methods include density gradients (DG), immunoaffinity, precipitation, size-exclusion chromatography (SEC), ultrafiltration, and microfluidics (71). Though we will not elaborate in this comprehensive review, we believe that groundbreaking methods can validate HSP70-exosomes as a novel tumor biomarker.

## 4 The role of eHSP70 in cancer progression

While delving into the application of eHSP70 as a tumor biomarker, many researchers have attempted to validate the feasibility of eHSP70 as a therapeutic target for cancer. This notion originates from numerous studies indicating the significant role of eHSP70 in several aspects of cancer progression, including proliferation and apoptosis, extracellular matrix (ECM) remodeling, epithelial-mesenchymal transition (EMT), angiogenesis, and immune environment modulation.

### 4.1 Proliferation and apoptosis

**Figure 2:**
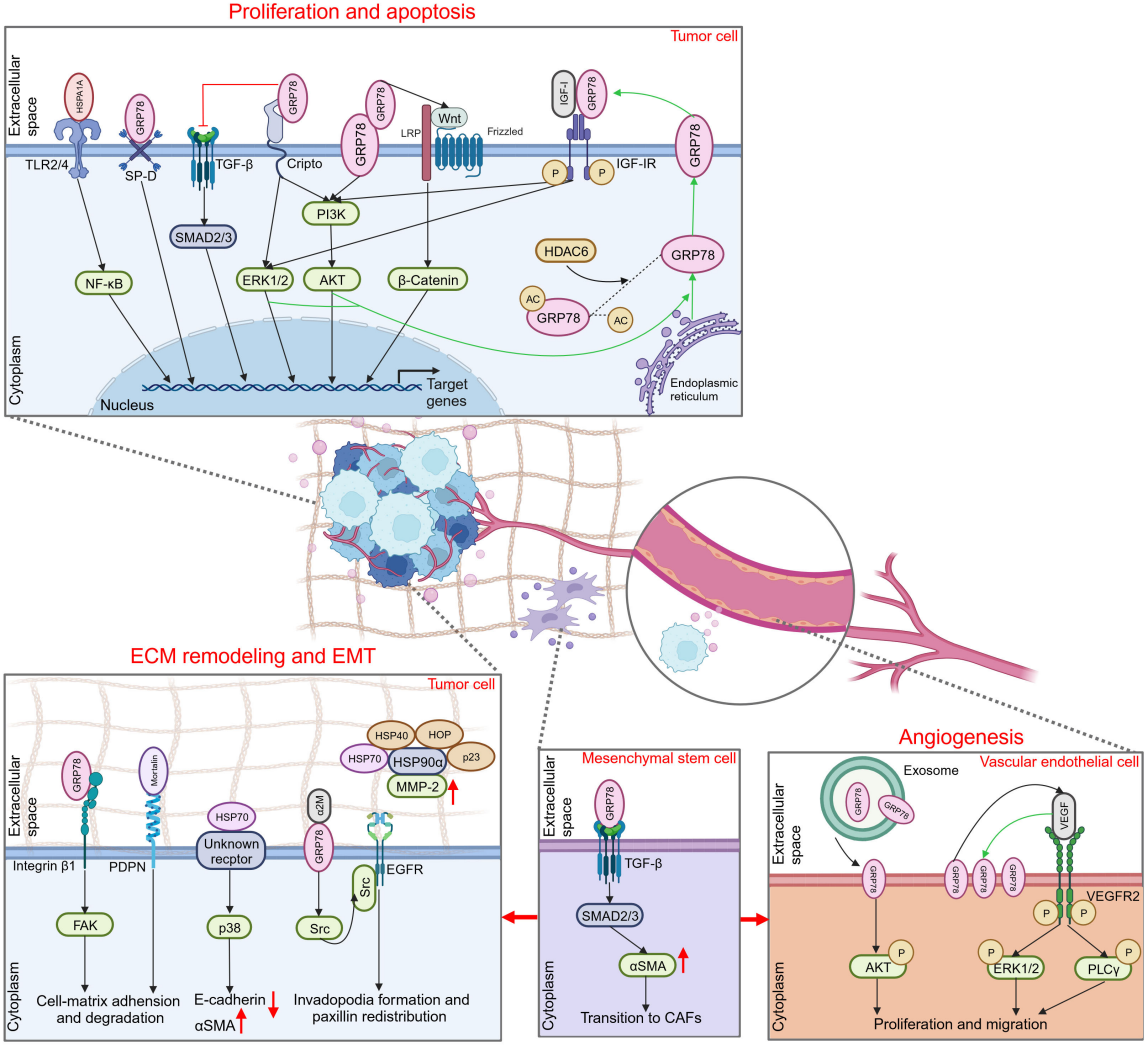
Promoting role of extracellular HSP70 in cancer progression. Through an autocrine way or tuning the behavior of different types of cells in the extracellular milieu, extracellular HSP70 is capable of enhancing cell proliferation, inhibiting cell apoptosis, and inducing ECM, EMT and angiogenesis to exert tumor-promoting function. TLR2/4, toll-like receptor 2/4; NF-κB, nuclear factor-κB; SP-D, surfactant protein-D; TGF-β, transforming growth factor β; SMAD2/3, small mother against decapentaplegic 2/3; ERK1/2, extracellular regulated protein kinases 1/2; PI3K, phosphoinositide 3-kinase; AKT, protein kinase B; LRP, lipoprotein receptor related protein; IGF-I, insulin-like growth factor I; IGF-IR, IGF-I receptor; HDAC6, histone deacetylase 6; ECM, extracellular matrix; EMT, epithelial-mesenchymal transition; FAK, focal adhesion kinase; PDPN, podoplanin; αSMA, α smooth muscle actin; α2M, α2-macroglobin; EGFR, epidermal growth factor receptor; MMP-2, matrix metalloproteinase-2; CAFs, cancer-associated fibroblasts; VEGF, vascular endothelial growth factor; VEGFR2, VEGF receptor 2; PLCγ, phospholipase C γ.

eHSP70 regulates the proliferation and apoptosis of numerous tumors, including colon cancer, hepatocellular carcinoma, breast cancer, endometrial cancer, glioma, and others (**Figure 2**) (72–76). The toll-like receptor (TLR) family constitutes a class of type I transmembrane glycoproteins that can recognize damage-associated molecular patterns (DAMPs) and participate in innate immunity (77). As a ligand for TLR2 and TLR4, extracellular HSPA1A potently stimulates proliferation and inhibits apoptosis in hepatocellular carcinoma cells via TLR2 and TLR4 signaling pathway and activation of nuclear factor-κB (NF-κB) (73). Cripto is a glycoprotein anchored to the membrane via glycosylphosphatidylinositol (GPI) that plays a pivotal role in tumor progression (78). It was found that the interaction of Cripto and GRP78 at the surface of cell membranes is crucial for cancer cell proliferation, potentially mediated via the activation of mitogen-activated protein kinase (MAPK)-phosphoinositide 3 kinase (PI3K) pathways and suppression of transforming growth factor β (TGF-β)-dependent phosphorylation of small mother against decapentaplegic 2/3 (SMAD2/3) (79, 80). In prostate cancer cells, disrupting the attachment of membrane GRP78 and surfactant protein-D (SP-D), a constituent of the collectin family, may interfere with the pro-tumorigenic role of extracellular GRP78 (81). Notably, GRP78 is not only secreted extracellularly but also interacts with the membrane-localized GRP78 as a receptor. This facilitates the proliferation of colon cancer cells by activating the PI3K/protein kinase B (AKT) and Wnt/β-catenin signaling, thereby introducing an innovative paradigm for the role of autocrine GRP78 in cancer progression (72).

In addition, some studies have focused on the intracellular to extracellular transport of HSP70 to elucidate its primary proliferating effect on tumor cells. Kim and colleagues revealed that inhibition of histone deacetylase 6 (HDAC6), a cytoplasmically localized deacetylase, can potentiate GRP78 acetylation, thereby attenuating the translocation of GRP78 to the cell surface via the PI3K/AKT signaling pathway. This event substantially inhibits the proliferation of cholangiocarcinoma and promotes its apoptosis, suggesting the pivotal role of membrane GRP78 in the progression of cholangiocarcinoma (82). In hepatoma cells, insulin-like growth factor I (IGF-I) similarly initiates GRP78 expression from the endoplasmic reticulum to the plasma membrane via the PI3K and MAPK pathways. This subsequently stimulates the phosphorylation and activation of the IGF-I receptor (IGF-IR) via membrane GRP78 and promotes IGF-I-mediated cellular proliferation and migration (83).

### 4.2 ECM remodeling and EMT

ECM is a complex network composed of macromolecules synthesized and secreted by cells that furnish biochemically and structurally supportive functions to the cellular component. This dynamic structure constantly undergoes a remodeling process, playing a significant role in cancer invasion and migration. In certain circumstances, this dynamic structure provokes another hallmark of cancer, EMT (84, 85). Several studies have illustrated that HSP70 can exert its effects on ECM remodeling and EMT via expression on the cell surface, and corresponding extracellular client proteins or membrane surface receptors associated with it have been sequentially uncovered (**Figure 2**). Cell surface GRP78 was identified to interact with the ECM adhesion molecule β1-integrin and mediate cell-matrix adhesion and ECM degradation by modulating focal adhesion kinase (FAK), thereby promoting colorectal cancer cell migration and invasion (86). Podoplanin (PDPN), which plays an important role in cell adhesion, is associated with mortalin released by oral squamous cell carcinoma cells on their cell surface (87). This suggests that extracellular mortalin potentially promotes tumor growth and invasion through PDPN-mediated pathways. In liver cancer, eHSP70 plays a critical role in regulating the EMT process (88, 89). Despite its unidentified receptor, eHSP70 has been confirmed to induce E-cadherin degradation and α smooth muscle actin (αSMA) overexpression by activating the p38/MAPK signaling pathway (88).

Activated α2-macroglobin (α2M*) is a natural circulating ligand of cell surface GRP78 (90). In conjunction with α2M*, membrane-associated GRP78 in hepatocellular carcinoma (HCC) cells is capable of interacting directly with Src, a tyrosine kinase, stimulating its phosphorylation and further facilitating the interaction between Src and epidermal growth factor receptor (EGFR). This effect can elicit the invasion and migration of HCC by promoting invadopodia formation and paxillin redistribution (91). Furthermore, eHSP70 may exert its function by orchestrating cells within the TME or interacting with other molecular chaperones. Cancer-associated fibroblasts (CAFs), a type of tumor-associated stromal cells, provide a favorable environment for the progression of malignant tumors through multiple mechanisms, such as ECM remodeling and angiogenesis (92). Peng and co-workers identified that GRP78 secreted by tumor cells can induce the differentiation of bone marrow-derived mesenchymal stem cells (BMSCs) to CAFs by activating the TGF-β/Smad signaling pathway (93). Another study reported that synergizing with HOP, HSP40, and p23 potentiates the activation of HSP90α on matrix metalloproteinase-2 (MMP-2), thereby enhancing breast cancer cell invasion and migration (94).

### 4.3 Angiogenesis

Angiogenesis is pivotal for nutritional supply and tumor metabolism, particularly in hypoxic conditions. Hypoxia can stimulate tumor cells to secrete vascular endothelial growth factor A (VEGFA) that binds to VEGF receptor 2 (VEGFR2) on neighboring vascular endothelial cells, thereby inducing the motility of endothelial cells and remodeling of ECM (95). The association between HSP70 and tumor angiogenesis was initially proposed by Dong and colleagues. They discovered that the microvessel density (MVD) of endogenous mammary tumors was significantly decreased in GRP78 heterozygous mice, with no effect on the MVD of normal organs (96). Subsequently, they investigated the role of GRP78 in the TME, revealing that the vascular formation was suppressed during the early phase of wild-type mammary tumors in GRP78 heterozygous mice. Similarly, the growth and metastasis of the tumor were profoundly inhibited (97). This finding indicated that GRP78 can contribute to tumor progression by promoting angiogenesis. Although the exact mechanism has not been elaborated, researchers believe that the extracellular expression of GRP78 may be involved in VEGF-induced angiogenesis. Subsequent studies verified these findings (**Figure 2**). Iha et al. observed that exosomes secreted by gastric cancer cells overexpressing GRP78 increased the proliferation and migratory capacity of co-cultured vascular endothelial cells, likely by the augmentation of AKT phosphorylation (98). Another study revealed that GRP78 was notably overexpressed on the membrane of human umbilical vein endothelial cells (HUVECs) following VEGF treatment, and ablation of GRP78 significantly suppressed VEGF-induced phosphorylation of phospholipase Cγ (PLCγ) and extracellular regulated protein kinases 1/2 (ERK1/2) and inhibited endothelial cell proliferation (99). These findings suggest that GRP78 not only contributes to angiogenesis through the secretion of tumor cells but also via epithelial cells. In addition, elevated HSP70 level was detected in tumor tissue samples of certain patients and was positively correlated with VEGF expression and MVD, substantiating the proangiogenic role of eHSP70 in tumors (100, 101).

### 4.4 Immune response

**Figure 3:**
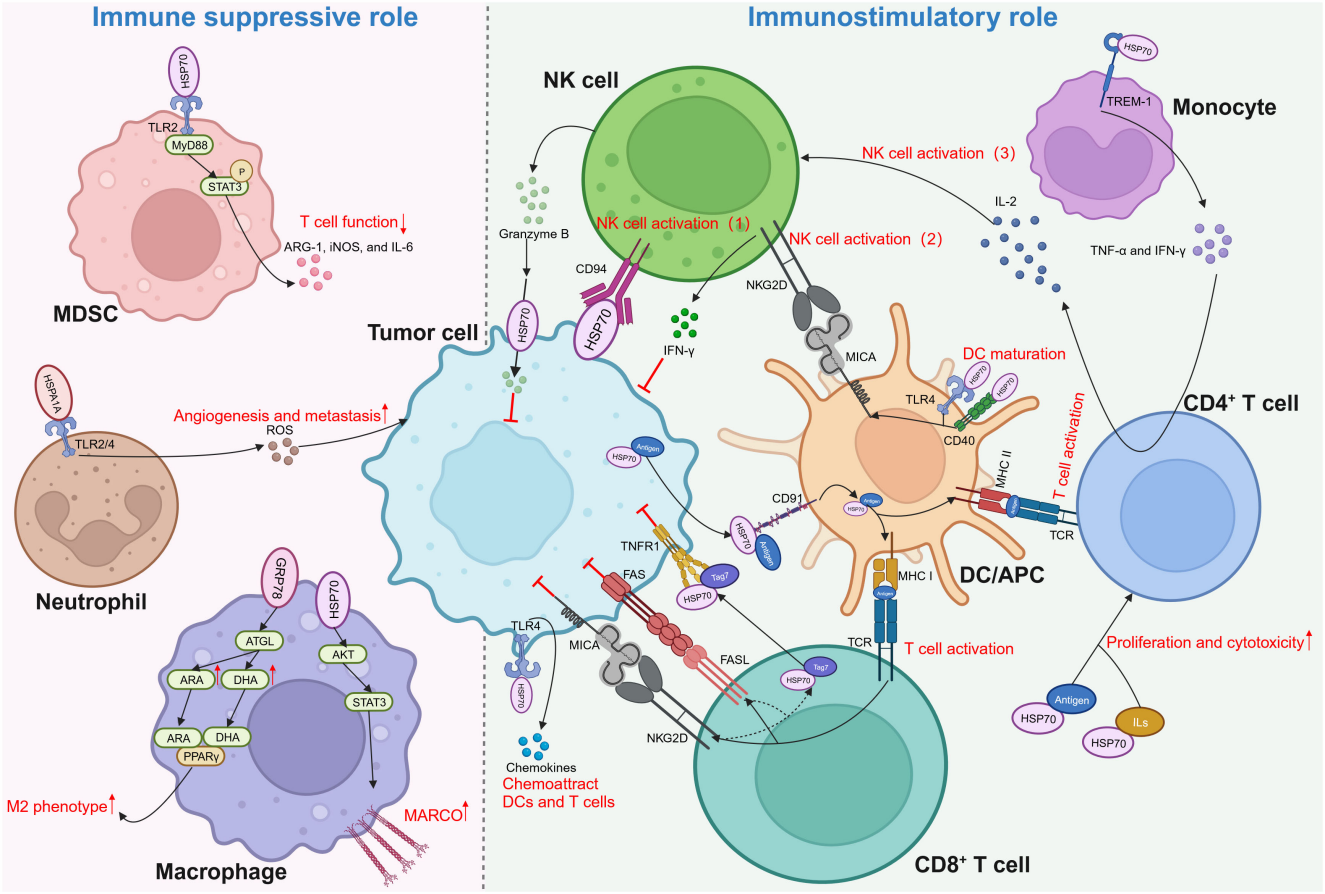
Dual role of extracellular HSP70 in immune response against cancers. Through activating different types of immune cells, extracellular HSP70 plays an immune suppressive role as well as immunostimulatory role. MDSC, myeloid-derived suppressor cells; TLR, toll-like receptor; MyD88, myeloid differentiation primary response gene 88; STAT3, p-signal transducer and activator of transcription 3; ARG-1, arginase-1; iNOS, inducible nitric oxide synthase; IL, interleukin; ROS, reactive oxygen species; ATGL, adipose triglyceride lipase; ARA, arachidonic acid; DHA, docosahexaenoic acid; PPARγ, peroxisome proliferator-activated receptor γ; AKT, protein kinase B; MARCO, macrophage receptor with collagenous structure; NK, natural killer; IFN-γ, interferon-γ; NKG2D, natural killer group 2 member D; TCR, T cell receptor; MHC, major histocompatibility complex; MICA, MHC class I chain-related protein A; TREM-1, triggering receptors expressed on myeloid cells-1; TNF-α, tumor necrosis factor-α; DC, dendritic cell; APC, antigen-presenting cell; TNFR1, tumor necrosis factor receptor 1; FAS, factor-associated suicide; FASL, FAS ligand.

The relationship between eHSP70 and immune response was first discovered to facilitate the release of pro-inflammatory cytokines from monocytes, such as interleukin (IL)-1β, IL-6, and tumor necrosis factor-α (TNF-α) (102–105). It engages monocytes via TLR2/4 in a CD14-dependent manner, stimulating the myeloid differentiation primary response gene 88 (MyD88)/interleukin-1 receptor-associated kinase (IRAK)/NF-κB signaling cascade to promote the production of pro-inflammatory cytokines (103). Other receptors, including CD40, CD36, and CD11b, were also found to mediate those effects, partly via the p38/MAPK pathway (104, 105). Subsequently, an increasing body of evidence indicated that eHSP70, contrary to its well-established oncogenic functions in other aspects of cancer biology, plays a paradoxical role in immune responses against cancer cells (**Figure 3**).

#### 4.4.1 Immune-suppressive function

Myeloid-derived suppressor cells (MDSCs) interact with HSP70 secreted in the form of exosomes by tumor cells, such as breast carcinoma, lung adenocarcinoma, ovarian carcinoma, and renal cell carcinoma cells (27, 106). In a TLR2/MyD88-dependent manner, exosomal HSP70 triggers p-signal transducer and activator of transcription 3 (STAT3) in MDSCs, thereby activating MDSCs and prompting the secretion of arginase-1 (ARG-1), inducible nitric oxide synthase (iNOS), and IL-6 (106). Reactive oxygen species (ROS) are important molecules that can enhance cancer progression, partly by stimulating the expression of VEGF and activating MMPs (107). Extracellular HSPA1A possesses the capacity to activate neutrophils and augment their generation of ROS by interacting with TLR2/4 on the surface of these innate immune cells (108). Macrophages demonstrate robust phagocytic proficiency in engulfing apoptotic cells. Specifically, during chemotherapy or radiation therapy, these cells are capable of phagocytosis and elimination of apoptotic tumor cells, demonstrating both anti-inflammatory and pro-tumor activities (109). A recent study revealed that HSP70 contained within EVs derived from colorectal cancer can enhance the phagocytosis ability of macrophages by upregulating macrophage receptors with collagenous structure (MARCO) via the AKT-STAT3 signaling pathways (110). Moreover, Tian et al. discovered that colorectal cancer cells can polarize macrophages into the M2 phenotype by regulating fatty acid metabolism in macrophages via secretory GRP78. By stabilizing adipose triglyceride lipase (ATGL), GRP78 can stimulate the synthesis of arachidonic acid (ARA) and docosahexaenoic acid (DHA) and enable them to interact with and activate peroxisome proliferator-activated receptor γ (PPARγ), resulting in the M2 polarization of macrophages (111).

#### 4.4.2 Immunostimulatory function

Nevertheless, eHSP70 is believed to be more inclined towards exhibiting an anti-tumor role through its substantial effect on various aspects of innate and adaptive immunity. In 2001, Multhoff and colleagues delineated that HSP70 located on the membrane of tumor cells can activate natural killer (NK) cells to evoke an innate immune response (112). Subsequent studies unveiled that HSP70 can be secreted extracellularly in exosomes or free soluble forms by various tumors, such as melanoma, colorectal cancer, and pancreatic cancer, to activate NK cells (113, 114). NK cells can induce perforin-independent apoptosis in HSP70 membrane-positive tumor cells by releasing serine protease granzyme B (115). This might be mediated by the interaction between eHSP70 and type C lectin receptor CD94 on the surface of NK cells (116–118). Additionally, two other mechanisms indirectly regulate NK cell activation by eHSP70. eHSP70 was reported to induce the expression of major histocompatibility complex (MHC) class I chain-related protein A (MICA) on dendritic cells (DCs). It activates NK cells and promotes the production of interferon-γ (IFN-γ) by binding to natural killer group 2 member D (NKG2D) on NK cells (119, 120). eHSP70 can also interact with the triggering receptors expressed on myeloid cells-1 (TREM-1) receptor on monocytes, resulting in the secretion of TNF-α and IFN-γ, stimulating IL-2 production by CD4^+^T cells, and activating NK cells (121). Moreover, HSP70 released by tumor cells into the TME can limit the conversion of a considerable proportion of monocytes to the pro-tumor phenotype (122).

The role of eHSP70 in antigen presentation by antigen-presenting cells (APCs) and activation of T cells is noteworthy. eHSP70 can induce the maturation of DCs by acting as a ligand for CD40 or TLR4 (123, 124). Through the TLR4 pathway, eHSP70 can increase the production of chemokines from tumor cells to recruit DCs and T cells (124). Specifically, eHSP70 can promote the cross-presentation of major histocompatibility complex (MHC) class I molecules by APCs to differentiate between antigens, which results in the activation of CD8^+^ T cells (125, 126). Activated CD8^+^ T cells can further release a cytotoxic Tag7-HSP70 complex, which induces tumor cell lysis via the tumor necrosis factor receptor 1 (TNFR1) (127). Moreover, secretion of the Tag7-HSP70 complex can be facilitated by the factor-associated suicide ligand (FasL) and NKG2D receptors on the surface of CD8^+^ T cells (128). HSP70-peptide complexes can also enter into the MHC class II presentation pathway to augment antigen-specific CD4^+^ T cell responses. The proliferation and cytotoxicity of CD4^+^ T cells can be enhanced by the HSP70-antigen complexes or HSP70 plus IL-2 or IL-7/IL-12/IL-15 (129, 130). Several studies found that HSP70 can bind to CD91 on APCs to mediate both MHC class I and II presentations (131–134). Other scavenger receptors, such as lectin-like oxidized low-density lipoprotein receptor-1 (LOX-1), were also shown to be HSP70-binding elements expressed on APCs (135–137). Interestingly, studies on HSP70 structure indicated that SBD plays a pivotal role in transporting antigens for binding to MHC class I, while attachment of antigens to the N-terminal results in efficient presentation on MHC class II (138, 139).

eHSP70 plays a pivotal role in cancer immume response. However, its role in the secretion of pro-inflammatory cytokines and activation of DCs and T cells remains controversial. Some studies suggested that lipopolysaccharide (LPS) contamination, not HSP70 itself, mediates this process (140–143). Therefore, numerous researchers avoided LPS contamination during their experiments, revealing that HSP70 itself can evoke these effects (105, 129). Regardless, additional studies are warranted to illuminate this ambiguity.

## 5 The role of eHSP70 in cancer treatment resistance

Beyond its role in cancer progression, eHSP70 can contribute to the treatment resistance of tumors. GRP78 was found to be released by bortezomib-resistant solid tumor cells. By promoting the phosphorylation of ERK and AKT and inhibiting p53-mediated induction of BOK and NOXA in HUVECs, GRP78 can protect these cells against the anti-angiogenic effects of bortezomib (144). Extracellular GRP78 is also involved in the tamoxifen resistance by binding to its partner protein CD44v at the COOH-terminal proline-rich region (145). A recent study showed that tumor-associated macrophages (TAMs) play vital functions in mediating eHSP70-induced chemoresistance in breast cancer cells. Exposure to epirubicin (EPI) induces breast cancer cells to secret HSP70, which modulates the intracellular expression of TGF-β and directly or indirectly amplifies the pro-tumor effects of TAMs (146). Furthermore, transfer of eHSP70 via small EVs enhances adriamycin (ADR) resistance through reprogramming energy metabolism in recipient cells. eHSP70 may be linked to cell stemness in cisplatin-induced cellular senescence, given that HSP70 levels on the plasma membrane increase after treatment with cisplatin (147, 148).

In addition to chemoresistance, eHSP70 is involved in resistance to other treatments. Upon radiation exposure, the membrane HSP70 expression level was significantly elevated in tumor cells (149). A study revealed that reduced HSP70 expression in the plasma membrane, rather than in the cytosol, diminishes the expression of γH2AX, caspase 3/7, and Annexin V, and decreases the post-irradiation survival rate of tumor cells. This elucidates that eHSP70 has the potential capability to diminish the lethal effects of radiotherapy on tumor cells (150). Cell surface GRP78 regulates the radio-resistance of glioma stem cells (GSCs) by activating the downstream β-site APP-cleaving enzyme 2 (BACE2), which upregulates NF-κB and C/EBPβ pathways (151). Recently, two pioneering studies by Theivanthiran and colleagues unveiled the pivotal role of eHSP70 in tumor adaptive resistance to anti-programmed death-1 (PD-1) immunotherapy. Through the intrinsic NOD-like receptor protein 3 (NLRP3)-HSP70-TLR4 axis, tumor cells can induce the recruitment of MDSCs after treatment with anti-PD-1 antibody. Specifically, eHSP70 regulates this process in an autocrine manner. NLRP3 activation triggers tumor cells to secret HSP70, which, in turn, interacts with TLR4 on the membrane of tumor cells, leading to the release of Wnt5a and upregulation of the CXCR2 ligand, CXCL5 (152). Furthermore, downstream of the TLR4/Wnt5a signaling pathway, initiated by eHSP70 in lung epithelial cells, may promote the recruitment of MDSCs in distal lung tissues, establishing a premetastatic niche that supports disease progression in response to anti-PD-1 immunotherapy (153).

## 6 Targeting eHSP70 for cancer treatment

Recognizing the significant role of eHSP70 in cancer progression, numerous studies assessed the feasibility of using eHSP70 as a potential therapeutic approach for treating cancer. However, owing to the dual role of eHSP70 in tumors, research efforts can be divided into two directions: inhibiting the tumor-promoting role of HSP70 on the membrane surface of tumor cells (**Table 2**), and developing HSP70-based vaccines to stimulate the immune response against tumor cells (**Table 3**).

**Table 2:** Selection of studies assessing cell surface HSP70 as a cancer therapeutic target.

Drug/antibody names	HSP70 types	Cancer types	Effects on tumors	Mechanisms	Refs
An antibody against the COOH-terminal domain of GRP78	GRP78	Prostate cancer, melanoma	Promotes tumor cell apoptosis *in vitro*	Upregulates p53 expression; downregulates the activation of AKT, ERK1/2, and p38 MAPK; Upregulates the JNK activity	(154, 155)
C38 (Anti-GRP78 COOH domain antibody)	GRP78	PDAC	Enhances tumor cell radiosensitivity	Curtails cell motility and invasion through inhibiting Rho-induced activation of YAP and TAZ	(156)
C107 (Anti-GRP78 COOH domain antibody)	GRP78	Melanoma	Inhibits tumor growth *in vivo*	—	(157)
MAb159 (Anti-GRP78 monoclonal antibody)	GRP78	Breast cancer, colon cancer, lung adenocarcinoma, small cell lung cancer, prostate cancer, leukemia	Inhibits tumor cell proliferation and promotes apoptosis *in vitro*; inhibits xenograft tumor growth and metastasis *in vivo*	Inhibits PI3K signaling	(158)
Heparin derivatives	GRP78	Prostate cancer	Inhibits xenograft tumor growth	Blocks autoantibodies binding to GRP78 and decreases tissue factor expression/activity	(159)
CHM-1	GRP78	NPC	Inhibits tumor cell proliferation and promotes apoptosis *in vitro*	Inhibits PI3K-AKT signaling by attenuating GRP78–p85α complex formation on the cell surface	(160)
FMBP	GRP78	Colorectal cancer	Inhibits tumor growth *in vitro* and *in vivo*	Promotes the intracellular accumulation of ROS by interfering with the activation of STAT3	(161)
ISM/BC71 peptide derived from ISM AMOP domain	GRP78	Breast cancer, melanoma	Inhibits tumor growth and promotes apoptosis *in vitro* and *in vivo*	Triggers tumor cell apoptosis by inducing mitochondrial dysfunction; triggers HUVEC apoptosis by activating caspase-8 and p53 signaling pathways	(162, 163)
A8 peptide aptamer	HSP70	Melanoma	Enhances tumor cell sensitivity to chemotherapy drugs (cisplatin/5FU)	Blocks HSP70/TLR2 association and the ability of tumor-derived exosomes to activate MDSCs	(27)

AKT, kinase-protein kinase B; ERK1/2, extracellular regulated protein kinases 1/2; MAPK, mitogen-activated protein kinase; JNK, c-Jun NH2-terminal kinase; PDAC, pancreatic ductal adenocarcinoma; YAP, Yes-associated protein; TAZ, tafazzin; PI3K, promoting phosphatidylinositol 3; CHM-1, 2’-fluoro-6,7-methylenedioxy-2-phenyl-4-quinolone; NPC, nasopharyngeal carcinoma; FMBP, a class III secretory peroxidase derived from foxtail millet bran; ROS, reactive oxygen species; STAT3, signal transducer and activator of transcription 3; ISM, isthmin; AMOP, adhesion-associated domain in MUC4 and other proteins; HUVEC, human umbilical vein endothelial cell; TLR2, toll-like receptor 2; MDSCs, myeloid-derived suppressive cells.

**Table 3:** Selection of preclinical studies and clinical trials assessing HSP70-based vaccines in cancer therapies.

Vaccine types	Vaccine components	Cancer types	Immunotherapy effects	Immune mechanisms	Refs
Preclinical studies					
Peptide-based vaccine	MAGE-HSP70 (MAGE1-MAGE3-MAGEn/HSP70, MAGE1-HSP70/SEA, TL-MAGE1-HSP70/SEA, MAGE-A1/HSP70)	HCC, melanoma	Prophylactic and therapeutic antitumor effects	Humoral and cellular immune responses	(164–167)
	PSCA-HSP70	Prostate cancer	Therapeutic antitumor effect	Humoral and cellular immune responses	(168)
	Mela-HSP70	Melanoma	Prophylactic antitumor effect	Cellular immune response	(169)
	HPV16 E7-HSP70	Cervical cancer	Prophylactic and therapeutic antitumor effects	Cellular immune response	(170, 171)
	EBV LMP2A-HSP70 (LMP2A (356–364) FLYALALLL-HSP70, LMP2A (426–434) CLGGLLTMV-HSP70)	Melanoma	Prophylactic and therapeutic antitumor effects	Cellular immune response	(172, 173)
	HSV VP22 (268–301)-HSP70	Lymphoma	Therapeutic antitumor effect	Cellular immune response	(174)
	MSLN-scFv/HSP70	Papillary ovarian cancer, malignant mesothelioma	Therapeutic antitumor effect	Cellular immune response	(175)
	A20-Id-ScFv/HSP70	Lymphoma	Prophylactic antitumor effect	Humoral and cellular immune responses	(176)
	Smlg-Id-ScFv/HSP70	CLL	—	Cellular immune response	(177)
TCL-based vaccine	DT-TCL-HSP70_407–426_	Breast cancer	Prophylactic antitumor effect	Humoral and cellular immune responses	(178)
	Dribble-HSP70_407–426_	Lung cancer	Therapeutic antitumor effect	Cellular immune response	(179)
DNA vaccine	HPV16 E7-HSP70	Cervical cancer, lung metastatic melanoma	Prophylactic and therapeutic antitumor effects	Cellular immune response	(180–185)
	DKK1-HSP70	Multiple myeloma	Prophylactic and therapeutic antitumor effects	Humoral and cellular immune responses	(186)
	AFP-HSP70	HCC	Therapeutic antitumor effect	Cellular immune response	(187, 188)
	PSCA-HSP70	Prostate cancer	Therapeutic antitumor effect	Humoral and cellular immune responses	(189)
	OVA_257–264_-HSP70	Lymphoma	Prophylactic antitumor effect	Cellular immune response	(190)
RNA vaccine	HPV16 E7-HSP70	Cervical cancer	Prophylactic antitumor effect	Cellular immune response	(191)
LV vector-based vaccine	TRP2-HSP70	Melanoma, breast cancer, glioblastoma	Therapeutic antitumor effect	Cellular immune response	(192)
DC vaccine	DCs pulsed with CEA_576–669_-HSP70L1	Colon cancer	Therapeutic antitumor effect	Cellular immune response	(193)
	DCs pulsed with HSP70-SPIONs and TCLs	Glioma	Therapeutic antitumor effect	Cellular immune response	(194)
	DCs pulsed with TCLs pulsed with HSP70_407–426_ and OK-432	HCC	Therapeutic antitumor effect	Cellular immune response	(195)
	DCs pulsed with HSP70-H22 tumor-peptide complexes and soluble CD40L	Hepatoma	Therapeutic antitumor effect	Cellular immune response	(196)
	DCs pulsed with tumor cell-derived HSP70-peptide complexes	HCC	—	Cellular immune response	(197)
NK cell vaccine	NK cells stimulated with HSP70-peptide TKD/IL-2 (+anti-PD-1 antibody)	Glioblastoma, colon carcinoma	Therapeutic antitumor effect	Cellular immune response	(198–200)
HUVEC vaccine	HUVEC-HSP70_407–426_	HCC	Prophylactic and therapeutic antitumor effects	Humoral and cellular immune responses	(201)
Vaccine combination therapy	AAV-BTLA vaccine + HSP70 vaccine	Lung metastatic melanoma	Prophylactic antitumor effect	Cellular immune response	(202)
	BTLA vaccine + HSP70 vaccine	Cervical cancer	Therapeutic antitumor effect	Cellular immune response	(203)
Clinical trials					
Peptide-based vaccine	HSP70-GPC3 vaccine (+LAG-3Ig/Poly-ICLC adjuvants)	Gastrointestinal cancer	Therapeutic antitumor effect	Cellular immune response	(204)

MAGE, melanoma-associated antigen gene; SEA, staphylococcal enterotoxins A; TL, tomato lectin; HCC, hepatocellular carcinoma; PSCA, prostate stem-cell antigen; HPV, human papillomavirus; EBV, Epstein-Barr virus; LMP2A, latent membrane protein 2A; HSV, herpes simplex virus; MLSN, mesothelin; scFv, single-chain antibody variable fragment; Id, idiotypic determinant; Smlg, surface membrane immunoglobulin; CLL, Chronic lymphatic leukemia; TCL, tumor cell lysate; DT, diphtheria toxin; Dribble, tumor-derived autophagome; DKK1, Dickkopf-1; AFP, alpha-fetoprotein; OVA, ovalbumin; LV, lentiviral; TRP2, tyrosinase-related protein-2; DC, dendritic cell; CEA, carcinoembryonic antigen; HSP70L1, HSP70-like protein 1; SPIONs, superparamagnetic iron oxide nanoparticles; NK, natural killer; TKD, TKDNNLLGRFELSG; IL-2, interleukin -2; PD-1, programmed death-1; HUVEC, human umbilical vein endothelial cell; AAV, adeno-associated virus; BTLA, B and T lymphocyte attenuator; GPC3, glypican-3; LAG-3, lymphocyte activation gene-3; Poly-ICLC, poly-riboinosinic-poly-ribocytidylic acid-poly-L-lysine carboxymethylcellulose.

### 6.1 Targeting cell surface HSP70

Misra and co-workers identified an antibody against the GRP78 COOH-terminal domain as a potential anti-cancer therapy, based on the results that it can promote tumor cell apoptosis by upregulating p53 expression and downregulating the activation of AKT, ERK1/2, and p38 MAPK (154, 155). Subsequently, several studies aimed to develop or evaluate monoclonal antibodies directed towards the COOH-terminal domain of GRP78. In pancreatic ductal adenocarcinoma (PDAC), the C38 monoclonal antibody was proven to curtail cell motility and invasion by inhibiting Rho-induced activation of Yes-associated protein (YAP) and tafazzin (TAZ). Using C38 can significantly enhance tumor cell radiosensitivity and increase the efficacy of radiation therapy (156). C107 monoclonal antibody was identified to have the ability of suppressing tumor growth as well. More crucially, its inhibitory effect appears superior to C38 in melanoma (157). Liu et al. reported another monoclonal antibody against GRP78 named MAb159, which exhibited notable anti-tumor effects in multiple tumor types, such as breast cancer, colon cancer, lung adenocarcinoma, small cell lung cancer (SCLC), prostate cancer, and leukemia. By blocking GRP78-induced activation of PI3K signaling, MAb159 can inhibit tumor growth and promote tumor metastasis in a mouse xenograft model (158).

In addition to monoclonal antibodies, some other drugs, compounds, or peptides were shown to disrupt the binding of HSP70 to its corresponding receptors or ligands on the cancer cell membrane, and remarkably inhibit its function. Treating with heparin derivatives can block autoantibodies binding to cell surface GRP78 and decrease tissue factor expression/activity, subsequently inhibiting tumor growth (159). By attenuating GRP78–p85α complex formation on the plasma membrane, 2’-fluoro-6,7-methylenedioxy-2-phenyl-4-quinolone (CHM-1) can reverse the inhibitory effect of apoptosis induced by the activation of PI3K/AKT signaling in nasopharyngeal carcinoma (NPC) cells (160). In colorectal cancer, the combination of a class III secretory peroxidase derived from foxtail millet bran (FMBP) with the NBD of cell surface GRP78 interferes with the downstream activation of STAT3, thus promoting the intracellular accumulation of ROS and cell grown inhibition (161). Isthmin (ISM) is a secretory 60-kDa protein that potently induces endothelial cell apoptosis. Two studies indicated that ISM or BC71 peptide derived from the ISM AMOP domain (adhesion-associated domain in MUC4 and other proteins) is capable of triggering the apoptosis of tumor cells and HUVEC by inducing mitochondrial dysfunction and activating caspase-8 and p53 signaling pathways, thus inhibiting tumor angiogenesis (162, 163). Furthermore, analyzing exosomes from various tumor samples indicated that A8 peptide aptamer can bind to the extracellular domain of membrane HSP70, effectively disrupting HSP70/TLR2 association and impairing the ability of tumor-derived exosomes to activate MDSCs. Given the fact that chemotherapy drugs, such as cisplatin and 5FU, augment the abundance of HSP70 exosomes, facilitating the activation of MDSCs and hindering the progression of anti-tumor immune response, a combination of A8 with cisplatin or 5FU can prevent MDSC activation (27).

Specifically, due to the high expression of HSP70 on the membrane of tumor cells, several studies have profiled eHSP70 as a molecular target for precision drug delivery. Chimeric antigen receptor T (CAR-T) cell immunotherapy is emerging as an effective cancer treatment; however, the application of CAR-T cell therapy in solid tumors remains limited. One possible reason is the absence of efficient tumor antigen targets (205). GRP78-directed CAR-T (GRP78-CAR-T) cells have shown strong anti-tumor efficacy in several solid tumors, such as pancreatic cancer, lung cancer, glioblastoma, and breast cancer, without obvious off-target effects or T cell infiltration in major organs (206–212). Moreover, several researchers have combined HSP70 binding peptide with other anti-cancer proteins or encased it within nanoparticles to precisely and effectively deliver chemotherapeutic drugs (213–220). For example, Farshbaf and co-workers loaded bortezomib with nanostructured lipid carriers (NLCs) modified with two proteolytically stable D-peptides, D8 and RI-VAP (Dual NLCs). Due to the high affinity of D8 for nicotine acetylcholine receptors on brain capillary endothelial cells and the high specificity of RI-VAP for binding to GRP78 on tumor cells, dual NLCs can pass the blood-brain tumor barrier with superior glioma-homing properties. They effectively deliver bortezomib and produce a potent anti-proliferative effect (221). The fusion of HSP70 tumor-penetrating peptide with nanoparticles can similarly be utilized to facilitate the delivery of novel hybrid iron oxide (Fe3O4), resulting in radiosensitization in triple-negative breast cancer cells (222). It is noteworthy that targeting cell surface HSP70 not only enhances drug delivery precision but also synergistically bolsters anti-tumor efficacy by suppressing HSP70 function. More studies, particularly clinical trials, are needed on the use of eHSP70 as a target for cancer treatment.

### 6.2 Developing HSP70-based vaccines

The development of HSP70-based vaccines is another key research topic. Although no relevant vaccination has been approved for clinical application, numerous studies have demonstrated robust anti-tumor effects of various types of vaccines that contain HSP70, effectively stimulating cellular or even humoral immune responses. Peptide-based vaccines are relatively easy to manufacture and usually composed of the HSP70 protein integrated with tumor-associated antigens (TAAs) or tumor-specific antigens (TSAs), such as melanoma-associated antigen gene (MAGE)-HSP70 (164–167), prostate stem-cell antigen (PSCA)-HSP70 (168), and Mela-HSP70 (169). Two studies produced a promising human papillomavirus 16 (HPV16) protein vaccine of E7-HSP70 that robustly induces E7-specific CD8^+^ T cell immune response and resulted in significant prophylactic and therapeutic effects against E7-expressing cervical cancer (170, 171). Other tumor-associated virus proteins, such as Epstein-Barr virus (EBV) latent membrane protein 2A (LMP2A) (172, 173) and herpes simplex virus (HSV) VP22 (174), were proved to generate effective cellular immune response after fusion with HSP70 protein in melanoma and lymphoma, respectively. Additionally, certain tumor markers were shown to possess limited immunogenicity but considerable specificity. Hence, linking the HSP70 protein to antibody fragments directed against them can dramatically enhance immunogenicity (175–177). Given these findings, a phase I study assessed the safety and efficacy of a novel vaccine comprising multi-human leukocyte antigen (HLA)-binding HSP70/glypican-3 (GPC3) peptides, a novel adjuvant combination of lymphocyte activation gene-3 (LAG-3) Ig, and poly-riboinosinic-poly-ribocytidylic acid-poly-L-lysine carboxymethylcellulose (Poly-ICLC) against metastatic gastrointestinal cancers. Seventeen patients received this vaccination therapy without dose-limiting toxicity, supporting the feasibility of this approach (204).

Compared to single TSA or TAA, tumor cell lysates (TCLs) encompass all self-tumor antigens and lack HLA-A2 restriction. Therefore, using them as vaccines can generate robust anti-tumor immune responses in a wide range of cancers (223). Fusion diphtheria toxin (DT) and two tandem repeats of HSP70_407–426_ with TCLs elicit strong humoral and cellular immune responses, leading to the growth inhibition of breast cancer (178). In addition, a tumor-derived autophagosome (Dribble) vaccine conjugated with HSP70_407–426_ significantly induced a higher expression of antigen-specific cytotoxic T lymphocytes (CTLs) (179). Genetic vaccines encompass DNA or RNA vaccines delivered by viruses or plasmids. Although no DNA vaccine has been commercialized globally to date, this particular strategy holds considerable promise. Studies indicated that the combination of HSP70 and HPV16 E7 (180–185, 191), Dickkopf-1 (DKK1) (186), alpha-fetoprotein (AFP) (187, 188), PSCA (189), or ovalbumin (OVA) (190) genes can induce potent immune responses against cancers. Lentiviral transfer of tyrosinase-related protein-2 (TRP2)-HSP70 gene is another efficacious and durable strategy in melanoma, breast cancer, and glioblastoma (192).

Several studies have prepared DC or NK cell vaccines stimulated with HSP70 to elicit an effective anti-tumor response by activating innate and adaptive immunity. Fusion protein CEA_576–669_-HSP70L1 can promote DC maturation and activate DC to produce cytokines, such as Il-12, IL-1β, and TNF-α, and chemokines, such as macrophage inflammatory protein-1α (MIP-1α) and MIP-1β. They subsequently elicit a potent CTL cytotoxicity against colon cancer (193). Immunization of glioma-bearing rats with DCs pulsed with superparamagnetic iron oxide nanoparticles (SPIONs) coated with HSP70 and tumor cell lysates (TCLs) resulted in a delayed tumor progression (194). In liver cancer, DCs pulsed with TCLs stimulated with HSP70_407–426_ and OK-432, HSP70-H22 tumor-peptide complexes and soluble CD40L, or tumor cell-derived HSP70-peptide complexes were all induced a cellular immune response (195–197). Furthermore, three studies demonstrated that NK cells stimulated with HSP70-peptide TKD/IL-2 may be a promising therapeutic approach for treating glioblastoma and colon carcinoma (198–200). A novel HUVEC-HSP70_407–426_ vaccine was reported to exert anti-angiogenesis effects by attenuating tumor-induced angiogenesis and reducing MVD of the intradermal tumor in mice (201). Other types of HSP70-based vaccines, like vaccine combination therapy, are also promising therapeutic approaches against cancer (202, 203). Interestingly, HSP70-based vaccines may induce acquired resistance of tumors to treatment. Geng et al. suggested that B7-H1 expressed by residual tumor cells may be the underlying mechanism. Blockade of B7-H1 by injection of a plasmid encoding the extracellular domain of PD-1 reversed this resistance and enhanced the therapeutic efficacy (224).

## 7 Conclusions

As a molecular chaperone, HSP70 is dynamically expressed and transported in response to stress stimuli, such as hypoxia, nutrient deficiency, immune stimulation, and therapeutic stress. Hence, the amount of HSP70 protein secreted by cancer cells is partly associated with the current state of the malignancy. This theory suggests that eHSP70 possesses the potential as a tumor biomarker. Analyzing clinical samples of cancer patients revealed that eHSP70 expression can be detected in tumor tissues or various body fluids, and variation in its expression level is associated with patients’ diagnosis, treatment response, and prognosis. Various types of cancers may show specific patterns of eHSP70 upregulation and downregulation that may distinguish them from healthy individuals and from patients with other non-cancerous conditions. Changes in eHSP70 expression may even discriminate one type of cancer from another. Moreover, the monitoring of therapeutic efficacy and potential recurrence can be facilitated by analyzing the expression levels of eHSP70 across several time points specific to each patient. Thus, it is not difficult to integrate artificial intelligence (AI) tools. For instance, quantitative proteomics and machine learning statistics can be integrated to construct a comprehensive cancer prediction network that depends on eHSP70. Nevertheless, the major challenge is how to detect eHSP70 expression real-timely, accurately, and sensitively. Aside from cell membrane eHSP70, the discernible forms of eHSP70 presently comprise the free soluble form, exosomal form, and autoantibodies against HSP70. Each one of these forms possesses advantages and disadvantages. The simplified detection method of free soluble HSP70 is advantageous; however, the short half-life of this form makes it challenging to precisely quantify its expression in a real-time manner. Exosomal HSP70 is more stable, but the current isolation technology for exosomes remains immature, and autoantibodies against HSP70 lack sufficient specificity and sensitivity. Therefore, overcoming these technological challenges can greatly facilitate the clinical use of eHSP70 as a tumor biomarker.

eHSP70 regulates the biological behavior of tumor cells. Not only can eHSP70 activate several signaling pathways in tumor cells in an autocrine manner but it can also tune the behavior of endothelial cells, immune cells, and other cells by interacting with extracellular components and membrane receptors. The diverse function of eHSP70 forms a complex network in the TME. On the one hand, it provides a favorable environment for tumor progression by promoting ECM, EMT, angiogenesis, and treatment resistance. On the other hand, it can act as a tumor-associated antigen to stimulate the activation and maturation of various immune cells, thereby provoking the immune response against tumor cells. Although some studies suggest its immunosuppressive role, most studies still support its immunostimulatory function. Given these findings, studies directly or indirectly targeted HSP70 on the membrane surface of tumor cells or developed HSP70-based vaccines. However, none of those therapeutic strategies have been approved for marketing. The inadequate safety and stability of HSP70-based tumor therapeutic strategies are challenging. On one hand, eHSP70 has different isoforms and is pivotal for preserving routine cellular activity. On the other hand, targeting distinct tumor-associated cells, such as cancer cells or immunocytes in the TME can potentially trigger different responses. Hence, understanding the structure of eHSP70, especially the SBD, and designing drugs based on this may be an effective way to solve the low specificity of eHSP70-targeting drugs. Furthermore, precisely targeting cell types that express eHSP70, such as precisely targeting HUVECs to inhibit angiogenesis promoted by eHSP70 or developing HSP70-pulsed DC vaccines, would make HSP70-based cancer treatment mores stable without affecting normal cell homeostasis or inducing unpredictable responses. Uncoupling the pro- and anti-tumorigenic effects of extracellular targets and membrane receptors of eHSP70 and selectively inhibiting their pathological activity can allow us to develop and assess HSP70-based anti-cancer therapies with greater clinical efficacies.

## Author contributions

BH: Writing – original draft, Investigation, Funding acquisition, Conceptualization. GL: Writing – original draft, Software, Conceptualization. KZ: Writing – review & editing, Supervision. GZ: Writing – review & editing, Validation, Supervision.
